# Reduced Severity in Patients With HIV-Associated Disseminated Histoplasmosis With Deep Lymphadenopathies: A Trench War Remains Within the Lymph Nodes?

**DOI:** 10.3389/fcimb.2020.598701

**Published:** 2021-02-08

**Authors:** Mathieu Nacher, Kinan Drak Alsibai, Antoine Adenis, Romain Blaizot, Philippe Abboud, Magalie Demar, Félix Djossou, Loïc Epelboin, Caroline Misslin, Balthazar Ntab, Audrey Valdes, Pierre Couppié

**Affiliations:** ^1^ CIC INSERM 1424, Centre hospitalier Andree Rosemon Cayenne, Cayenne, French Guiana; ^2^ DFR Santé, Université de Guyane, Cayenne, French Guiana; ^3^ Department of Pathology, Centre hospitalier Andrée Rosemon, Cayenne, French Guiana; ^4^ Department of Dermatology, Centre hospitalier Andrée Rosemon, Cayenne, French Guiana; ^5^ Service des Maladies Infectieuses et Tropicales, Centre hospitalier Andrée Rosemon Cayenne, Cayenne, French Guiana; ^6^ Laboratory, Centre hospitalier Andrée Rosemon Cayenne, Cayenne, French Guiana; ^7^ UMR Tropical Biome and Immunopathology, Université de Guyane, Cayenne, French Guiana; ^8^ Service de Médecine, Centre hospitalier de l’Ouest Guyanais, Saint Laurent du Maroni, French Guiana; ^9^ Département d’Information Médicale, Centre hospitalier de l’Ouest Guyanais, Saint Laurent du Maroni, French Guiana; ^10^ Equipe Opérationnelle d’hygiène hospitalière, Centre hospitalier Andrée Rosemon Cayenne, Cayenne, French Guiana

**Keywords:** HIV, disseminated histoplasmosis, liposomal amphotericin B, French Guiana, lymph node

## Abstract

**Background:**

Disseminated histoplasmosis is a major killer of patients with advanced HIV. It is proteiform and often hard to diagnose in the absence of diagnostic tests. We aimed to describe disseminated histoplasmosis with lymphadenopathies in French Guiana and to compare survival and severity of those patients to patients without lymphadenopathies.

**Methods:**

A retrospective cohort study was performed on data records collected between January 1, 1981 and October 1, 2014.

**Results:**

Among 349 cases of disseminated histoplasmosis 168 (48.3%) had superficial lymphadenopathies and 133(38.1%) had deep lymphadenopathies. The median LDH concentration, ferritin concentration, TGO concentration, and WHO performance status were lower among patients with deep lymphadenopathies than those without deep lymphadenopathies. There was a significant decrease in the risk of early death (<1 month) among those with deep lymphadenopathies relative to those without (OR=0.26 (95%CI=0.10–0.60), P=0.0006) and in the overall risk of death (OR=0.33 (95%CI=0.20-0.55), P<0.0001). These associations remained strongly significant after adjusting for time period, CD4 counts, age, delay between beginning of symptoms and hospital admission, antifungal and antiretroviral treatment.

**Conclusions:**

The present data show that in patients with advanced HIV and disseminated histoplasmosis, the presence of deep lymphadenopathies is associated with fewer markers of severity and a lower risk of death. To our knowledge it is the first study to show this. The presence of deep lymphadenopathies is hypothesized to reflect the patient’s partially effective defense against *H. capsulatum*.

## Introduction

*Histoplasma capsulatum* is a dimorphic ascomycete that grows in soil and bird and bat guano. After spores are inhaled, it transforms into a pathogenic yeast and replicates within macrophages which can transport the yeast from the lungs to any organ (Horwath et al., 2015). The efficient clearance of *Histoplasma* yeasts requires the activation of macrophages through the Th1 response. The recognition of *H. capsulatum* by dendritic cells and macrophages promotes the differentiation and recruitment of Th1 cells. However, this process fails in immunocompromised individuals who therefore are at risk for disseminated infection. Antigen presenting cells migrate to the nearest lymph node where the antigen they express along with MHCII molecules can be presented to a variety of circulating naïve lymphocytes that can eventually become activated and proliferate. Humans usually have about 500 lymph nodes, which are divided into groups and are more concentrated near the trunk (Standring, 2016). As secondary lymphoid organs, lymph nodes have a central role in the development of adaptive immunity against pathogens. During infections, they may become enlarged and palpable. Ever since the beginning of the HIV epidemic, lymph node pathology has been known to be an important consequence of human immunodeficiency virus (HIV) infection. The central role that lymphoid tissues play in HIV pathogenesis has been suggested by the structural and functional alterations induced by HIV. (Dimopoulos et al., 2017) Enlarged lymph nodes are common among HIV-infected patients and there may be several causes, infections by *Mycobacterium tuberculosis*, Lymphoma, Castelman syndrome, Kaposi syndrome, or HIV itself. Hence, before being called HIV, the virus responsible for AIDS was called Lymphadenopathy Associated Virus. Another infecting pathogen that can cause lymphadenopathies is *Histoplasma capsulatum*.

Since the 1980s, HIV has spread in French Guiana, a French overseas territory between Brazil and Suriname. A distinct feature of patients with advanced HIV in French Guiana is that disseminated histoplasmosis is the most frequent AIDS-defining infection and cause of death. This epidemiological fact has been known by dermatologists since the 1980s, and then spread in the medical community further accelerated by fungal culture from a variety of tissue samples, which increasingly allowed identifying the fungal pathogen (Nacher et al., 2019; Morote et al., 2020). Lymphadenopathies are common and easily accessible sites to sample tissue to diagnose histoplasmosis (Huber et al., 2008).

In this context, we aimed to describe our experience regarding disseminated histoplasmosis with lymphadenopathies in French Guiana and to compare survival and severity of those patients to patients without lymphadenopathies.

## Methods

### Study Design

A retrospective multicentric study was performed on patients with confirmed disseminated histoplasmosis included between January 1st, 1981 and October 1st, 2014. The cohort is not funded and its updating it will require the availability of staff sufficiently trained to collect all these data until 2020.

### Study Population

Co-infections with HIV and histoplasmosis were enrolled in the Histoplasmosis and HIV database of French Guiana. The inclusion criteria were as follows: confirmed HIV infection; first proven episode of histoplasmosis [EORTC/MSG criteria (De Pauw et al., 2008)]; and age >18 years.

### Study Design

This database was created in 1992. It included incident cases of HIV-associated histoplasmosis in the three hospitals of French Guiana. Epidemiological, clinical, paraclinical, immunovirological and therapeutic data were collected on a standardized case record form until October 2014. Hospitalized incident cases of HIV-associated histoplasmosis were included. Sex, age, place of birth, symptoms on admission, clinical entrance examination, immunovirological status, standard biological examinations, medical imaging, mycology, pathology, treatment received, and survival data during the study period were collected. Lymphadenopathies were classified according to size (<=2 cm, between 2 and 5 cm, and > 5 cm) and superficial (palpable) or deep lymphadenopathies, which were not palpable but visible in patients having benefitted from medical imagery (ultrasound, CT-scanner).

### Statistical Analysis

STATA^©^ (College Station, Texas, USA) was used. Quantitative variables were described using medians and interquartile ranges, they were compared between groups with or without superficial lymphadenopathies, or between groups with or without deep lymphadenopathies using ranksum non-parametric tests or Student’s t-test, where appropriate. For qualitative variables, Chi2 or Fisher tests were computed comparing the proportions between those with and without lymphadenopathies. We also compared those with lymphadenopathies >2 cm to those without lymphadenopathies >2 cm. Multivariate logistic regression was used to adjust for potential confounders. Modeling included variables that significantly differed between those with and without lymphadenopathies. Model fit was verified using the Hosmer Lemeshow goodness of fit test. Kaplan Meier curves were computed and the Log Rank test was used to compare patients with and without lymphadenopathies. Patients lost to follow-up were right-censored at the date of last visit. Statistical significance was set at P<0.05.

### Ethical and Regulatory Aspects

The research was approved by the Comité Consultatif pour le Traitement de l’Information pour la Recherche en Santé (CCTIRS) (number 10.175 bis, 10/06/2010), the French National Institute of Health and Medical Research institutional review board (CEEI INSERM) (IRB0000388, FWA00005831 18/05/2010), and the Commission Nationale Informatique et Libertés (CNIL) (number JZU0061856X, 07/16/2010).

## Results

**Figure 1:**
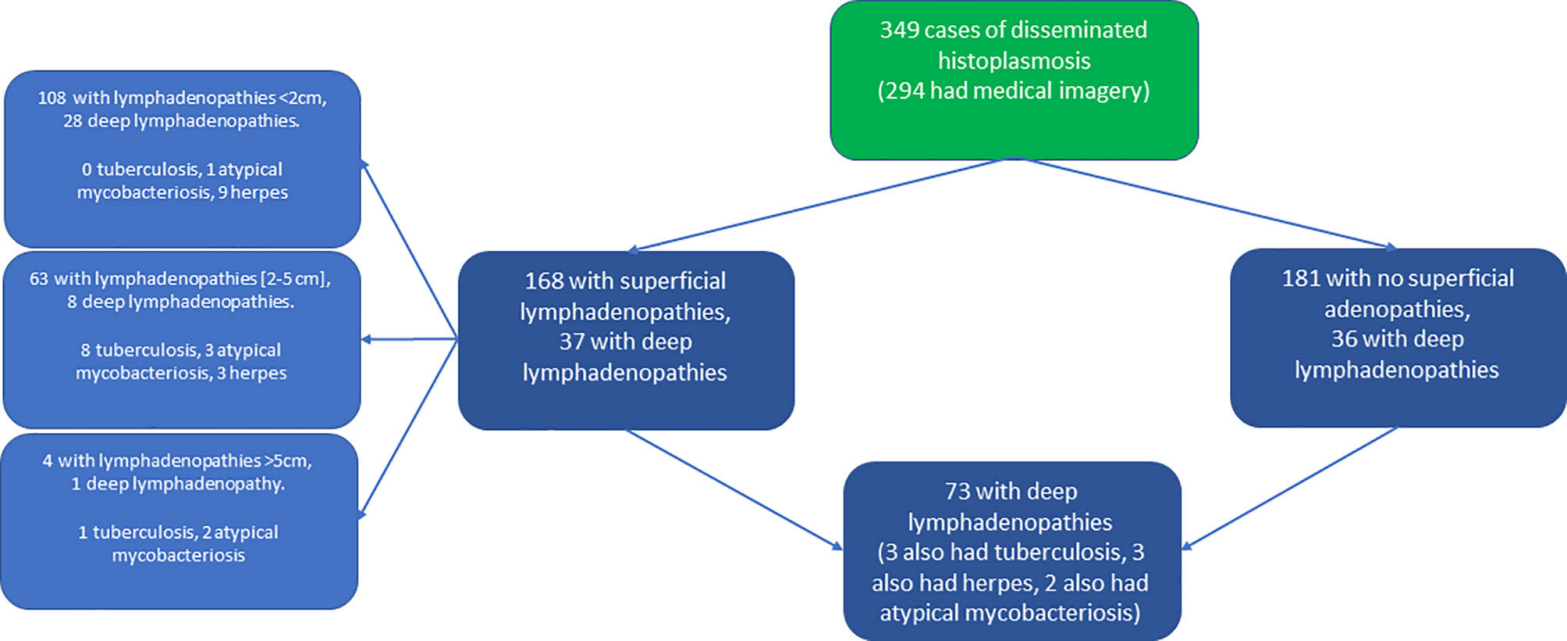
Flowchart stratifying patient counts by presence of superficial or deep lymphadenopathies.

Among 349 cases of disseminated histoplasmosis between January 1, 1981 and October 1, 2014, 168 (48.3%) had superficial lymphadenopathies and 133 had deep lymphadenopathies (38.1% if considering all patients, but 133/294 (45.2%) when only considering patients having had CT-scans or ultrasonography). **Figure 1** shows a flowchart breaking down the types and sizes of lymphadenopathies, and the persons with concomitant tuberculosis, atypical mycobacteriosis, and chronic herpes.

### Superficial Lymphadenopathies

Among the 168 patients with superficial lymphadenopathies, 108 (64.3%) had lymphadenopathies <2 cm, 63 (37%) between 2 and 5 cm, and 4 (2.4%) >5 cm (**Table 1**). Patients with lymphadenopathies >2 cm were less likely to have pulmonary signs than those without lymphadenopathies >2 cm (**Table 2**). Patients with lymphadenopathies >2cm were also less likely to have had thoracic or abdominopelvic CT-scans, digestive endoscopy and bone marrow aspiration than those without lymphadenopathies >2 cm (**Table 2**). The median neutrophil count was higher among patients with lymphadenopathies >2cm than those without lymphadenopathies >2 cm (**Table 2**). The median platelet count was higher among patients with lymphadenopathies >2cm than those without lymphadenopathies >2 cm (**Table 2**). There were no significant differences between those with or without superficial lymphadenopathies for hemoglobin, ferritin, C-reactive protein, or LDH (data not shown).

**Table 1:** Superficial and deep lymphadenopathies in human immunodeficiency virus (HIV)-infected patients with disseminated histoplasmosis.

Variable	n/N	Percentage
*Presence of superficial^†^ lymphadenopathies*	168/349	48
Superficial lymphadenopathies<2 cm	108/168	64
*cervical lymphadenopathies<2 cm*	60/100	60
*supraclavicular lymphadenopathies <2 cm*	11/100	11
*axilary lymphadenopathies <2 cm*	38/100	38
*inguinal lymphadenopathies<2 cm*	49/100	49
Medium size superficial lymphadenopathies (2–5 cm)	63/168	37
*cervical lymphadenopathies (2–5 cm)*	23/35	65
*supraclavicular lymphadenopathies (2–5 cm)*	6/35	17
*axilary lymphadenopathies (3–5 cm)*	19/35	54
*inguinal lymphadenopathies (3–5 cm)*	8/35	23
Large superficial lymphadenopathies (>5 cm)	4/168	2
*cervical lymphadenopathies>5 cm*	2/4	50
*supraclavicular lymphadenopathies >5 cm*	0/4	0
*axilary lymphadenopathies >5 cm*	2/4	50
*inguinal lymphadenopathies>5 cm*	1/4	25
*Deep^‡^ lymphadenopathies*	133/349	38
Mediastinal lymphadenopathies on chest Xray	4/132	3
Deep lymphadenopathies on abdominopelvic ultrasonography	82/234*	35
*Abdominal*	76/83	91
*Celiomesenteric*	32/83	38
*interaortico-caval*	18/83	21
*Lombo-aortic*	14/83	16
*hepatic hilus*	12/83	14
*Illiac*	6/83	7
*Latero-caval*	5/83	6
Deep lymphadenopathies on abdominal-pelvic CT-scanner	60/98*	61
*Abdominal*	40/61	65
*Celiomesenteric*	32/61	52
*Retroperitoneal*	24/61	39
*Lombo-aortic*	13/61	21
*interaortico-caval*	12/61	19
*Illiac*	12/61	19
*Hepatic hilus*	5/61	8
*Latero-caval*	3/61	4
Deep lymphadenopathies on thoracic CT-scanner	37/100*	37
*Mediastinal*	31/37	83
*Axillary and supraclavicular*	9/37	24
*Hilar*	7/37	18
*Sub carinal*	2/37	5
*Barety’s space*	1/37	2

*Number of procedures performed; ^†^superficial lymphadenopathies are palpable during clinical examination; ^‡^deep lymphadenopathies are not palpable and only visible through medical imagery.

**Table 2:** Comparisons between patients with and without large superficial lymphadenopathies (>2 cm), and with and without deep lymphadenopathies.

	Superficial^†^ lymphadenopathies>=2cm N=67	No superficial^†^ lymphadenopathies>2cm N=282	P*
Delay between symptoms onset and diagnosis (median [IQR]) days	143 [51–261]	42 [20–188]	0.0004
Pulmonary symptoms and signs	36.5	60.9	0.002
Thoracic CT Scan (%)	14.3	44.7	<0.0001
Abdominopelvic CT Scan (%)	11.1	40	<0.0001
Bone marrow aspiration (%)	6.3	26.7	0.001
Endoscopy (%)	28.5	44.7	0.03
Neutrophils (median [IQR]) per mm3	2,240 [1,380–3,050]	1,525 [6–2,726]	0.001
Platelets (median [IQR]) per mm3	194,500 [101,000–262,500]	106,000 [384–213,500]	0.001
CD4 count (median [IQR]) per mm3	46 [19–112]	26 [60–103]	0.003
Proportion receiving liposomal amphotericin B induction (%)	34.9	52	0.03
	**Deep** **lymphadenopathies^‡^** **N=133**	**No deep** **lymphadenopathies^‡^** **N=216**	P*
LDH (median [IQR]) U/L	349 [261–540]	538 [320–1,309]	<0.0001
Ferritin (median [IQR]) ng/ml	1082 [388–1,908]	1347 [641-5,449]	0.01
TGO (median [IQR]) IU	50 [31–75]	60 [35–119]	0.01
WHO performance status>2 (%)	41.5	51.8	<0.001
Proportion with ultrasonographic hepatomegaly (%)	56.2	75.5	0.02

*chi square test for proportions, rank sum test for medians; ^†^superficial lymphadenopathies are palpable during clinical examination; ^‡^deep lymphadenopathies are not palpable and only visible through medical imagery.

Patients with superficial lymphadenopathies were less likely to receive presumptive antifungal treatment than those without superficial lymphadenopathies, 80.9% vs 88.8%, P=0.04. There was no significant difference in median CD4 count (32 (IQR=12-73) vs 31 (IQR=14-65), P=0.7) between those with and without superficial lymphadenopathies, respectively. The delay between diagnosis of histoplasmosis and the beginning of symptoms was longer among patients with superficial lymphadenopathies >2 cm than in those without. However, these patients had greater CD4 counts than those without superficial lymphadenopathies >2 cm (**Table 2**). There was no difference in the proportion of patients receiving amphotericin b (liposomal or deoxycholate) whether they had superficial lymphadenopathies or not (45.4%, vs. 41.8, P=0.5). However, those with superficial lymphadenopathies >2 cm were less likely to receive amphotericin B induction therapy than those without (**Table 2**).

Although there seemed to be a lower proportion of deaths within 1 month after antifungal therapy among those with superficial lymphadenopathies than those without, the difference was not significant (11.3% vs 17.2%, P=0.11). Similarly, for all deaths, there was a non-significant trend for fewer deaths in the superficial lymphadenopathies (36.9% vs 45.5%, P=0.1).

Among persons explored with medical imagery, persons with superficial lymphadenopathies were more likely to also have deep lymphadenopathies (OR=1.87 (95%CI=1.18-2.97), P=0.004.

### Deep Lymphadenopathies

Regarding deep lymphadenopathies, the median LDH concentration was lower among patients with deep lymphadenopathies than those without deep lymphadenopathies (**Table 2**). The median ferritin concentration was lower among patients with deep lymphadenopathies than those without deep lymphadenopathies (**Table 2**). The median TGO concentration was lower among patients with deep lymphadenopathies than those without deep lymphadenopathies (**Table 2**). Ultrasonographic measurement of hepatomegaly showed that the proportion of patients with hepatomegaly was lower among patients with deep lymphadenopathies than in those without deep lymphadenopathies (**Table 2**).

When looking at the WHO performance status there was less general condition alteration among those with deep lymphadenopathies (**Table 2**).

### Time and Lymphadenopathies

**Figure 2:**
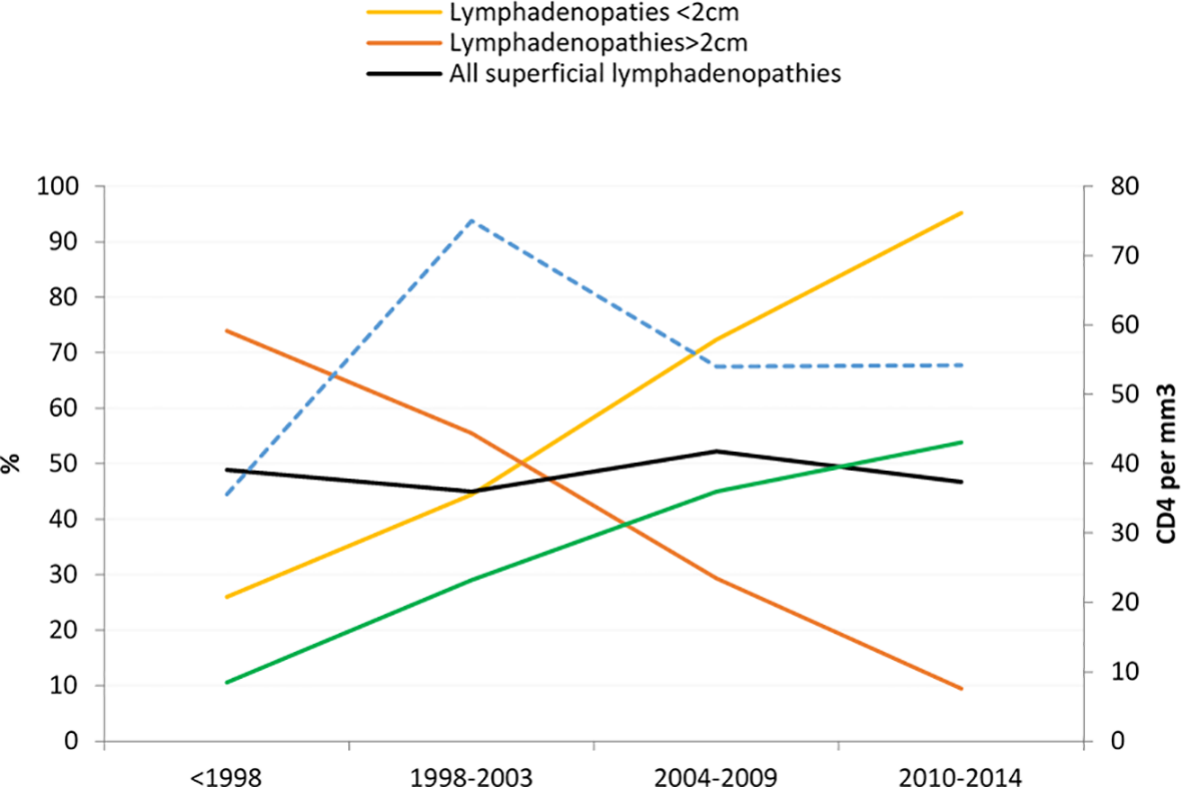
Evolution of the proportion of patients with lymphadenopathies by size.

When comparing the proportion of patients with lymphadenopathies between four time-periods (<1998, 1998-2003, 2004-2009, 2010-2014) there was no significant difference between the proportion of patients with superficial lymphadenopathies during different time periods. However, **Figure 2** shows that over time the proportion of lymphadenopathies >2 cm decreased whereas those < 2 cm increased (P for linear trend <0.0001), and among those having benefitted from medical imagery the proportion of deep lymphadenopathies increased (P<0.0001). There was a strong negative correlation between time period and the duration between symptoms’ onset and hospital admission (Spearman’s rho −0.57, P<0.0001).

**Figure 3:**
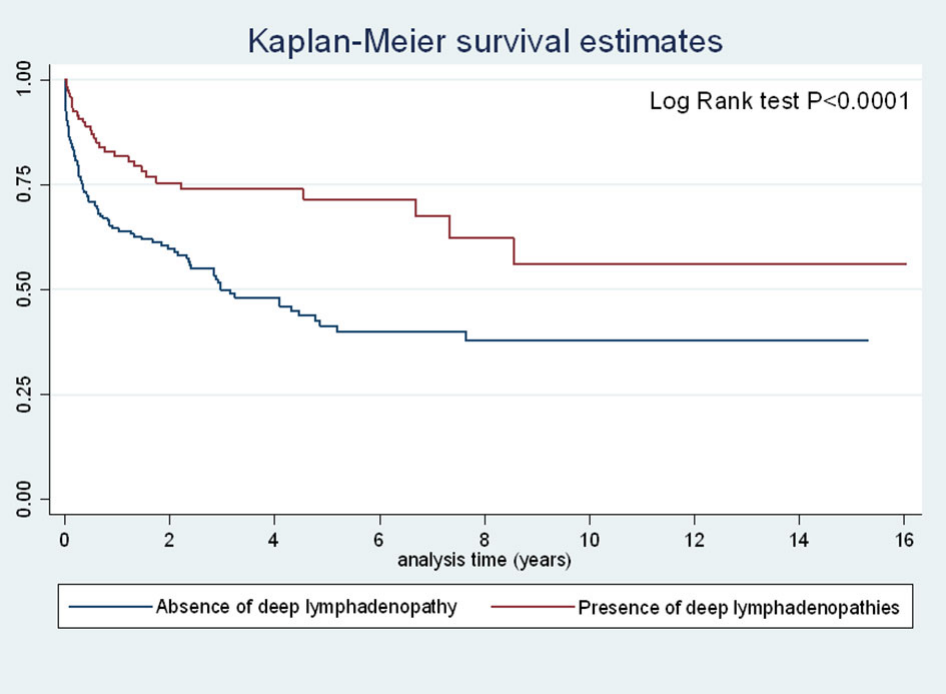
Incidence of death among patients with disseminated histoplasmosis stratified by the presence of deep lymphadenopathies.

**Figure 4:**
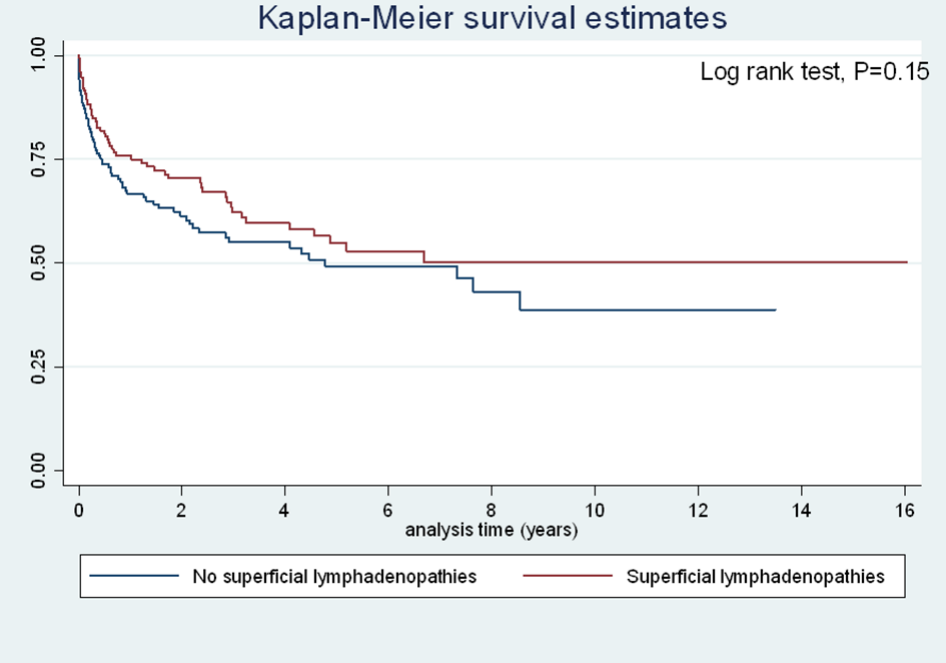
Incidence of death among patients with disseminated histoplasmosis stratified by the presence of superficial lymphadenopathies.

There was a significant decrease in the risk of early death (<1 month) among those with deep lymphadenopathies relative to those without (OR=0.26 (95%CI=0.10–0.60), P=0.0006) and in the overall risk of death (OR=0.33 (95%CI=0.20–0.55), P<0.0001). These associations remained strongly significant after adjusting for time period, CD4 counts, age, delay between beginning of symptoms and hospital admission, antifungal and antiretroviral treatment. **Figures 3** and **4** show the Kaplan Meier curves for survival by deep lymphadenopathies and superficial lymphadenopathies considering the onset of symptoms as origin, the date of death, and censoring at the date when the patient was last seen. This amounted to 850 person-years of follow up. Both curves suggest that survival was greater among those with lymphadenopathies but the log Rank was not significant for superficial lymphadenopathies. The incidence rate of death among patients without superficial lymphadenopathies was 17.5 per 100 person-years, and for those with superficial lymphadenopathies it was 12.3 per 100 person-years. The incidence rate of death among patients without deep lymphadenopathies was 19.5 per 100 person-years, and for those with deep lymphadenopathies it was 8.4 per 100 person-years.

Overall, there were no differences between those with or without lymphadenopathies and transmission modes, history of opportunistic infections, antiretroviral treatment, nationality, sex (data not shown). There were 9 (12.3%) concomitant tuberculosis cases in those with lymphadenopathies and 9(14.3%) concomitant tuberculosis cases in those without lymphadenopathies, P=0.7. There were 6 (8.2%) concomitant cases of atypical mycobacterial infection in those with lymphadenopathies and 1(1.6%) concomitant case of atypical mycobacterial infection in those without lymphadenopathies, P=0.08. There were 12 (16.4%) concomitant chronic herpes cases in those with lymphadenopathies and 8(12.7%) concomitant chronic herpes cases in those without lymphadenopathies, P=0.5. Performing the above comparisons between patients with and without superficial or deep lymphadenopathies after excluding persons with concomitant tuberculosis, mycobacteriosis and chronic herpes did not change the observed differences (data not shown). There were 23/55 positive direct examinations of lymph node samples and 43/53 positive cultures of lymph node samples, and 39/63 positive pathological examinations of lymph node biopsies.

## Discussion

Superficial lymphadenopathies were very common among patients with disseminated histoplasmosis and 19% of all patients had lymphadenopathies >2 cm. Among patients having benefitted from ultrasonography or CT-scanner, 45% had deep lymphadenopathies. Patients with superficial lymphadenopathies seemed less severe that those without superficial lymphadenopathies but this failed to reach statistical significance. However, patients with deep lymphadenopathies were definitely less severe than patients without deep lymphadenopathies. After adjusting for several potential confounders, they were less likely to die, at one month and at any temporal horizon than patients without deep lymphadenopathies. Consistently with this finding, markers that can serve as a proxy for severity (Couppie et al., 2004) were more favorable than those of patients without deep lymphadenopathies. Hence, LDH, Ferritin, and TGO levels were significantly lower, and platelet counts were higher in those with deep lymphadenopathies. They were also less likely to have ultrasonographic hepatomegaly than those without deep lymphadenopathy, and the WHO performance status score was lower (indicating less alteration of the patient’s general condition) among patients with deep lymphadenopathies than among those without deep lymphadenopathies.

Patients with superficial lymphadenopathies >2 cm were less likely to have further explorations than those without, presumably because they were less likely to have respiratory problems, to have leukocyte or platelets cytopenias than patients without lymphadenopathies >2 cm. In addition, the attending physicians may also have focused on the tangible anomaly at hand and thus, they were less intensively explored. Patients with superficial lymphadenopathies were less likely to receive presumptive antifungal treatment than those without superficial lymphadenopathies. The delay between diagnosis of histoplasmosis and the beginning of symptoms was longer among patients with superficial lymphadenopathies>2cm than in those without such lesions. These patients had greater CD4 counts than those without superficial lymphadenopathies >2 cm. Patients with superficial lymphadenopathies >2 cm were less likely to receive amphotericin B induction therapy than those without, which is also an indication they were less severe.

There were a number of potential limitations. Lymphadenopathies are frequent in HIV-infection, a frequency that may vary depending on the proportion of advanced immunosuppression or the pathogen ecosystem (Cortez et al., Sep-Oct; Kamana et al., 2010; Hadadi et al., 2014). Among patients with concomitant opportunistic infections that can cause lymphadenopathy we did not observe any significant differences between those with or without superficial lymphadenopathies, but the total numbers of coinfections were relatively small. It was hence often not possible to determine for sure the cause of enlarged lymphadenopathies when there was no aspiration or biopsy for fungal diagnosis. Diagnosis was reached in different ways and the fungal load was not known. It is possible to suspect a bias where those with superficial lymphadenopathies would be diagnosed earlier because of the externality of lymph nodes. However, the fact that the delay between symptoms’ onset and diagnosis was longer in those with superficial lymphadenopathies >2 cm than in those without, and that their CD4 count was higher was consistent with the observation that deep lymphadenopathies were associated with less mortality. Finding deep lymphadenopathies depended on actually looking for them using medical imagery. This raises the question of considering that those who did not get an ultrasound or a CT-scan could wrongly be considered as not having deep lymphadenopathies. However, when excluding those who did not have ultrasonographic or CT-scanographic explorations, the findings did not change at all. Over 34 years many things have changed, HIV testing, histoplasmosis diagnostic capacity and awareness, antifungal treatment, and HIV treatment (Nacher et al., 2020). All this may introduce biases, but our observations of reduced death remained very significant after taking into account confounders such as age, the duration of symptoms before admission, antifungal and antiretroviral treatment, and CD4 count. Therefore, we are confident in the conclusion that disseminated histoplasmosis with deep lymphadenopathies is statistically less severe that when there are no deep lymphadenopathies. An intriguing finding was the fact that superficial lymphadenopathies >2 cm became significantly less frequent over time, whereas lymphadenopathies <2 cm became more frequent, while median CD4 count remain stable all along. This was perhaps linked to the gradual decline of delays between the onset of symptoms and hospitalization and treatment, which left less time for lymphadenopathies to grow.

The corpus of knowledge of pathologists also brings an interesting way to consider our results. Histopathological lesions in histoplasmosis reflect the host reaction against *H. capsulatum* and are usually classified into four categories including tuberculoid, anergic, mixed, and sequelae types. The tuberculoid form generally corresponds to a low inoculation and effective tissue response of the host. In this form, *H. capsulatum* are usually few in number and are located in the cytoplasm of macrophages (intracellular). The anergic form is usually seen in HIV patients and shows little or no tissue response but abundant intracellular and extracellular yeast. In anergic forms the local macrophages remain inactive. The mixed form represents an intermediate form between the tuberculoid and the anergic type. Finally, in the sequelae type, scarring fibrosis is predominant and inflammation is mild. In this form, *H. capsulatum* yeasts are rare and may correspond to a relapse case or possible reactivation.

In our Histoplasmosis pathology experience, 31 patients had histological confirmation of lymph node histoplasmosis by the presence of *H. capsulatum* yeasts on lymph node biopsies or surgical specimens. Interestingly and consistently with the clinical outcome of this study the granulomatous inflammatory reaction of tuberculoid type was the most frequent (24/31, 77.4%) which usually corresponds to effective tissue response (Drak Alsibai et al., 2020). Among these 24 granulomatous cases, histological variants perfectly mimicking tuberculosis with epithelioid granulomas, multinucleated giant cells and caseous necrosis were found in 10 cases, followed by less typical epithelioid granulomatous variants where giant cells and necrosis were absent in eight cases, then by forms with predominant macrophage infiltration in six cases. The remaining seven lymph nodes showed non-specific hyperplastic lymphadenitis.

Lymph nodes function as an active innate barrier that can help improve patients’ defenses against the spread of infection. Furthermore, they can be actively modulated to rapidly recruit additional cells in response to a response default. In the normal lymph node, the macrophages reside in the subcapsular sinus and medullary sinus. Lymphatic fluid brings pathogens and antigen-presenting cells to lymph nodes macrophages (Bogoslowski and Kubes, 2018). In infectious diseases, the granuloma is the result of a complex interaction between the infectious agent and a wide range of inflammatory cells (macrophages, lymphocytes T and B, etc…) and biological mediators (cytokines, interleukins, chemokines, growth factors, etc…).

Granuloma formation is promoted by a close relationship between activated macrophages that strongly express major histocompatibility complex (MHC) class II molecules and CD4+ T helper cells 1 (Th1) (James, 2000). Macrophages may develop into epithelioid cells and can also fuse to form multinucleated giant cells. Th1 recognize protein peptides presented to them by antigen presenting cells carrying MHC II molecules. Th1 subsequently induces interleukin-1 on macrophages that promote granuloma formation. The overstimulation of Th1 cells compared to T helper cells 2 (Th2) leads to cell hyperactivity, tissue destruction and granuloma reaction. Instead, when the Th2 is over-stimulated, it causes an anergy and apoptosis. The activation of Th1 and Th2 lymphocytes is controlled by B7-CD28/CTLA-4 stimulation pathways (James, 2000).

Our convergent findings strongly suggest that patients who have significantly enlarged superficial and deep lymphadenopathies are less severe. An enlarged lymph node is a sign that there is a battle going on in the lympnode, where circulating lymphocytes are confronted with antigen presenting cells. For the proliferation to take place the immunocompromised host must still have some capacity to mount part of a response. An analogy could be immune reconstitution syndromes, for which one of the common presentations are rapidly growing and at times compressive lymphadenopathies where the reconstitution of the immune system is “visible” in the lymphnodes (Nacher et al., 2006; Melzani et al., 2020). Even though the patient has low CD4 counts, the enlarged lymphadenopathies in patients with disseminated histoplasmosis may thus testify than an active “front line” is remaining. This lower severity is of course relative, and disseminated histoplasmosis is still a very serious disease even with deep lymphadenopathies as shown by the incidence rate of 8.4 deaths per 100 person-years. The patterns of dissemination of this proteiform disease are still obscure, in some disseminating to certain organs and in others in different organs (Couppié et al., 2019). The interplay between inoculum, immunity, personal factors, random factors results in a great variety of clinical presentations, requiring different microbiologic diagnostic approaches; all this lead to the difficulty of this diagnosis in the absence of antigen detection tests (Wheat, 1994).

In conclusion, the present data showed that in patients with advanced HIV and disseminated histoplasmosis, the presence of deep lymphadenopathies- and perhaps of superficial lymphadenopathies- was associated with fewer markers of severity and a lower risk of death. To our knowledge it is the first study to show this.

## Data Availability Statement

The raw data supporting the conclusions of this article will be made available by the authors after permission from the commission nationale informatique et libertés, without undue reservation.

## Ethics Statement

Written informed consent was obtained from the individual(s) for the publication of any potentially identifiable images or data included in this article.

## Author Contributions

Conceptualization, MN. Methodology, MN. Formal analysis, MN. Investigation, LE, RB, AV, AA, PA, FD, MD, CM, BN, KA, PC. Data curation, AA, PC. Writing—original draft preparation, MN. Review and editing, LE, RB, AV, AA, PA, FD, MD, CM, BN, KA, PC. All authors contributed to the article and approved the submitted version.

## Conflict of Interest

The authors declare that the research was conducted in the absence of any commercial or financial relationships that could be construed as a potential conflict of interest.
